# Improved Swiss-rolling method for histological analyses of colon tissue

**DOI:** 10.1016/j.mex.2022.101630

**Published:** 2022-02-09

**Authors:** Julie Le Naour, Léa Montégut, Adrien Joseph, Kévin Garbin, Erika Vacchelli, Guido Kroemer, Jonathan G. Pol, Maria Chiara Maiuri

**Affiliations:** a Centre de Recherche des Cordeliers, Inserm U1138, Team “Metabolism, Cancer & Immunity” Equipe labellisée par la Ligue contre le cancer, Université de Paris, Sorbonne Université, 15 rue de l'Ecole de Médecine, Paris 75006, France; b Metabolomics and Cell Biology Platforms, Gustave Roussy Cancer Campus, Villejuif, France; c Faculty of Medicine Kremlin Bicêtre, Université Paris Saclay, Le Kremlin Bicêtre, France; d CHIC (Histology, Imaging and Cytometry Center), Centre de Recherche des Cordeliers, Paris, France; e Hôpital Européen Georges Pompidou, AP-HP, Paris, France

**Keywords:** Intestinal murine tissue, Swiss-rolling technique, Ex vivo method, Histological tissue preparation, EtOH, Ethanol, PBS, Phosphate-buffered saline, PFA, paraformaldehyde

## Abstract

Since the introduction of the Swiss-rolling technique by Reilly and Kirsner in 1965, various methodological approaches have been developed for histological analyses of intestinal tissues. Here, we describe an improved protocol for the processing of freshly harvested murine colons that can be extended to other intestinal tissues. With simple tools, this technique allows to tightly wrap the organ throughout the whole length and to keep it in place before fixation, avoiding excessive stiffness of the tissue. Unlike the original method which relies on frozen samples, processing of the biological samples right after resection preserves epitopes integrity for subsequent immunohistochemical analyses. Ultimately, this method provides a reproducible workflow to capture the entire colon length in a unique histological section in order to assess several features such as intestinal inflammation and tumorigenesis.

• Easily include freshly isolated tissues

• Shorten preparation time using a few affordable tools

• Prevent unrolling and preserve tissue integrity

## Graphical abstract

**Figure:**
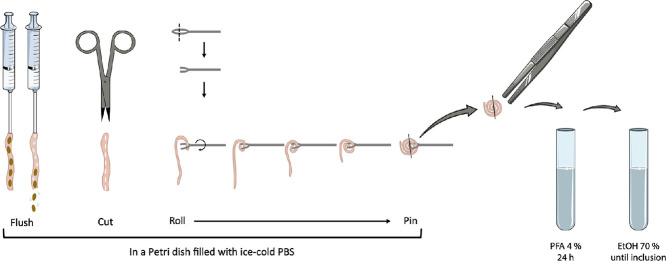

Specifications Table

**Table:** 

Subject Area:	Biochemistry, Genetics and Molecular Biology
More specific subject area:	Histology
Method name:	Minuten-pinned colon Swiss-rolling
Name and reference of original method:	1. Reilly RW, Kirsner JB. Runt Intestinal Disease. Lab Invest. 1965;14:102–107. 2. Moolenbeek C, Ruitenberg EJ. The “Swiss roll”: a simple technique for histological studies of the rodent intestine. Lab Anim. 1981;15(1):57–59. doi:10.1258/002367781780958577
Resource availability:	*n/a*

## Material

### Animals

Six- to 8-week-old female C57Bl/6 J mice were purchased from Envigo or bred in the local animal facility. Mice were maintained in the animal facility in specific pathogen-free conditions in a temperature-controlled environment with 12-h light/12-h dark cycles and received food and water *ad libitum*. Animal experiments followed the Federation of European Laboratory Animal Science Association (FELASA) guidelines and were in compliance with EU Directive 63/2010. Protocol #24,973–2,020,040,413,162,969 v3 was approved by the Charles Darwin Ethical Committee (French Ministry of Research).

### Histology materials

- 150-µm diameter minuten pins (n°15 from Sphinx can be purchased from taxidermy stores).
- Bond RX multiplex immunohistochemistry stainer (Leica).
- HistoCore Arcadia H - Heated Paraffin Embedding Station (Leica).
- HistoCore Arcadia C - Cold Plate (Leica).
- Large-eye tapestry needles (Size 20, Bohin).
- Microtome (#RM2245, Leica).
- Tissue processor (#ASP300, Leica).

### Materials

- 1 mL Syringe injeckt-F 1 ml (#9,166,017 V, B.Braun).
- 5 mL polystyrene round-bottom tube (#352,054, Corning).
- 500 mL beaker.
- Forceps (#11,650–10, #11,650–11 or #11,602–14, Phymep).
- Petri dishes (#BP94A-01, Corning).
- Plastic gavage needles (#FTP-20–38, Phymep).
- Scissors (#14,958–09, #14,959–09, #14,084–08 or #14,085–08, Phymep).
- Tissue processing/embedding cassette (#M505–2, Simport).

### Reagents

- Anti-CD3, clone D4V8L (#99940S, Cell Signaling).
- BOND Dewax solution (#AR9222, Leica).
- DAB Substrate Kit (# 34,002, ThermoFisher).
- Eosin-Y aqueous (#6,766,010, MM France).
- Ethanol absolute (#20,821.310 or #20,821.365 VWR).
- Haemalum-Shorr staining solution (#720–0330, VWR)
  Paraffin (#39V2001 or #39,601,006, Leica).
- Phosphate buffered saline (#100,123, Gibco).
- Pierce™ 16% formaldehyde (w/v), methanol-free (#28,908, Thermo Fisher Scientific).
- Rabbit HRP PowerVision Kit (#PV6119, Leica).
- Saffron (natural), flower (#720–0184, VWR).
- Sub-X clearing medium (#3,803,672, Leica).

## Method

### Swiss rolls[1], [2]

***Day 1***

In the lab:

- Preparation of 4% paraformaldehyde (PFA): dilute 10 mL of Pierce™ 16% formaldehyde (w/v), methanol-free (#28,908, Thermo Fisher Scientific) in 30 mL of phosphate-buffered saline (PBS).
- Prepare 500 mL of 70% ethanol (EtOH): dilute 350 mL absolute EtOH in 150 mL of water.
- Turn large-eye tapestry needles (size 20–22) into two-tinned fork by cutting the eye in half with scissors.

In the animal facility:

- Sacrifice the mouse by cervical dislocation.
- Wet the abdominal fur of the mouse.

*Note:* This step will prevent deposition of hair on internal tissues after incision

- Make a small V-shaped incision in the upper part of the abdomen.
- Place round tip scissors in the first cut and make a xypho-pubic incision of the skin and peritoneum.
- Disentangle intestinal tracts.
- Retrieve the colon (or any intestinal tissue of your convenience).
- Place it in a Petri dish filled with ice cold PBS.
- Remove surrounding fat tissue from the organ.
- Extensively wash the inner part of the intestine tract by gently flushing ice-cold PBS through it using a needle with a gavage needle.
- Repeat the procedure to get rid of all feces.
- Place the colon in a clean Petri dish filled with ice cold PBS.
- Cut it open longitudinally.

*Tip****:*** Use ball tip scissors at this step to avoid damaging the colon lumen

- Gently flatten the colon with the luminal side facing up.
- Slide the proximal extremity of the colon between the two tines of the homemade fork, then slowly roll towards the distal end.

**Fig. 1:**
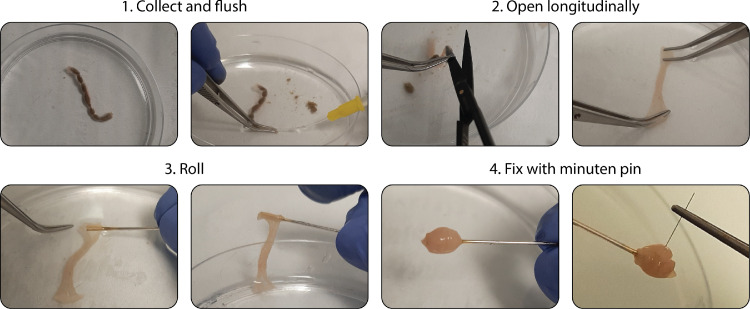
Workflow of the improved Swiss-rolling technique for intestinal tissue. Representative photographs of the rolling process. The four main sequential steps are depicted: (1) colon washing, (2) dissection, (3) rolling and (4) fixation.

*Tip:* Use the side of the Petri dish as a guide to stay perfectly aligned (Fig. 1)

- Fix the roll with a minuten pin using small forceps. The pin has to be placed between the two tines of the fork in order to go through all colon layers.

*Tip:* Only manipulate minuten pins with small forceps as they are extremely thin (150-µm ∅) and easily pierce gloves

- Place the Swiss roll in a tube containing 4 mL of 4% PFA at 4 °C for 24 h (Fig. 1).

*Day 2*

- After fixation, transfer the roll in an inclusion cassette and keep it in 70% EtOH at 4 °C for at least 2 h until paraffin embedding.

*Tip:* Label the cassette with a pencil to avoid EtOH dissolution of the ink

### Histology

*Samples preparation*

(1) Impregnate swiss rolls using ASP300 tissue processor

- Dehydrate tissues with five successive baths of absolute EtOH for 50 min each.
- Clarify tissues in two successive baths of SubX for 30 and 40 min, respectively.
- Imprenate tissues in three baths of paraffin for 35 min each at 60 °C.

(2) Embed samples in paraffin using HistoCore Arcadia H and C

- Fill the appropriate mold with liquid paraffin.
- Place the surface of the section at the bottom of the mold to obtain the desired orientation.
- Place the mold on the cold region of Arcadia H to fix the sample.
- Place the cassette over the mold and check that it is within the paraffin.
- Place the cassette and mold on the cold region of Arcadia C.
- Unmold the paraffin bloc.

(3) Prepare sections

- Perform 3-μm thick sections with microtome.
- Dry at 37 °C until the next day.
- Dewax slides in 2 baths of Bond Dewax solution for 30 s at 72 °C and one bath at room temperature.
- Rehydrate slides in 3 baths containing EtOH 100% each bath for a few seconds.

*Hematoxylin, eosin, and saffron (HES) coloration*

- Stain nucleus with Mayer's Hemalun staining solution for 4 min 30 s.
- Stain cell cytoplasm with 1% aqueous eosin for 2 min.
- Dehydrate slides in 3 baths of EtOH at 70%, then 100%, for 1 min each.
- Stain collagen with 1% alcoholic safran for 3 min 30 s.
- Place the slides in 4 successive baths of 100% EtOH for 1 min each.
- Place the slides in 3 successive baths of xylen for 1 min each.
- Coverslipping with Pertex mounting medium.

*CD3 staining*

- Incubate the slide in epitope retrieval solution 2 (pH 9) for 20 min at 100 °C.
- Block endogenous peroxidase with 3% hydrogen peroxide in deionized water, for 10 min.
- Rinse well with Bond wash buffer.
- Incubate the slides with anti-CD3 antibody (1/100, clone D4V8L, #99,940, Cell Signaling) for 1 h at room temperature.
- Rinse well twice with Bond wash buffer.
- Incubate the slides with the “ready to use” Poly-HRP anti-Rabbit IgG (#PV6119, Leica) for 30 min at room temperature.
- Rinse well twice with buffer.
- Wash in buffer for 5 min.
- Reveal using 3,3′-Diaminobenzidine (DAB, # 34,002, ThermoFisher) 5 min at room temperature.

Instruction for DAB preparation: mix one drop of DAB chromogen and 1 mL substrate solution

- Counterstaining with Hematoxylin staining solution for 5 min.
- Rinse well with deionized or tap water.
- Coverslipping with Pertex mounting medium at room temperature.

*Slides scan and visualization*

**Fig. 2:**
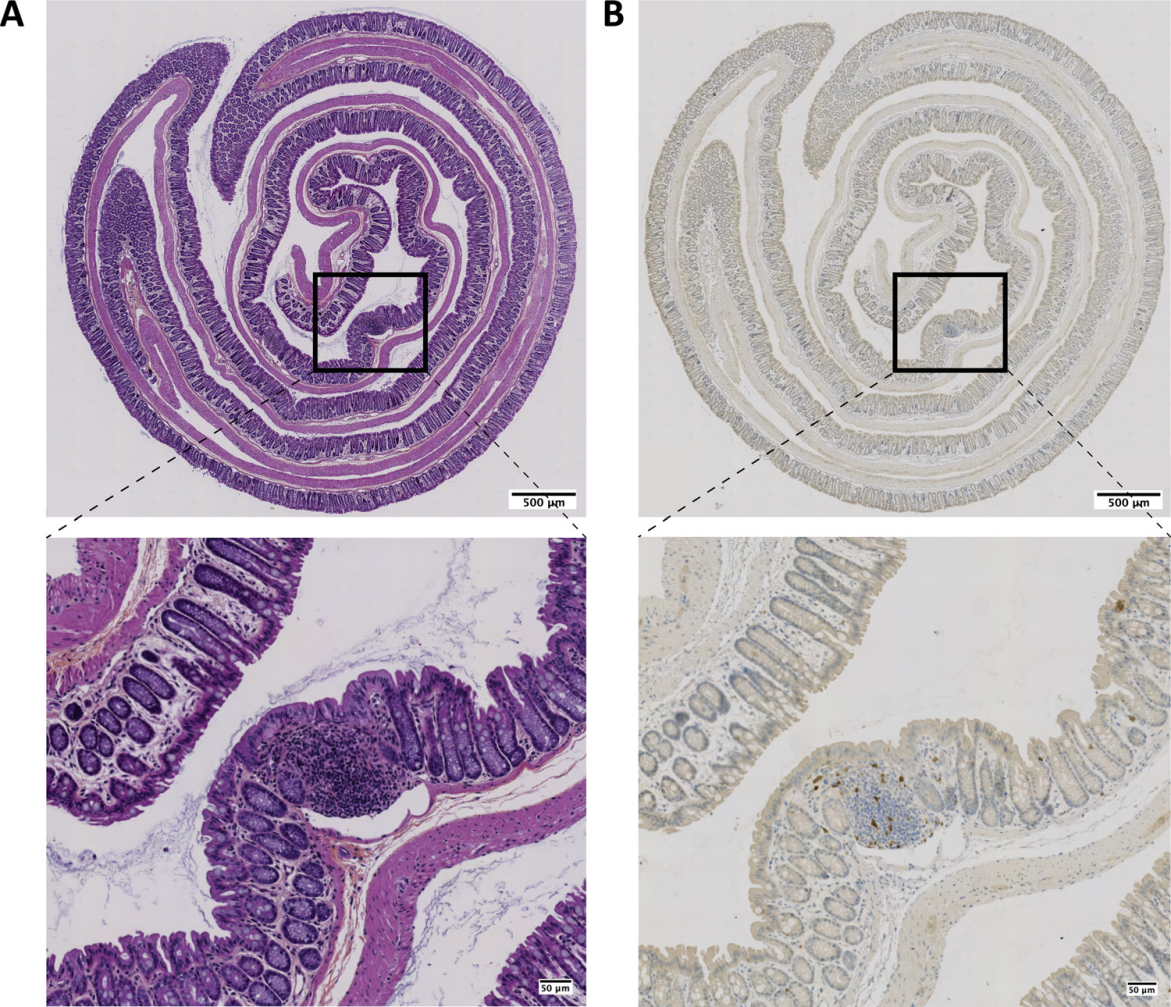
Representative images of (immuno) histochemical stainings of colon Swiss-rolls. **A** Hematoxylin, eosin, and saffron (HES) coloration. B CD3 staining revealed with 3,3′-Diaminobenzidine (DAB) with respective zooms on a tertiary lymphoid structure taken with a Plan-Apochromat x20/0.8 objective with a 0.22 µm/pixel numerical resolution.

- Scan each slide using Zeiss Axio Scan Z1 slide scanner.
- Visualize using the QuPath-0.2.3 software (Fig. 2).

## Declaration of Competing Interest

JGP is named as inventor on patents for cancer vaccination involving an oncolytic rhabdovirus. These patents have been licensed to Turnstone Biologics of which JGP is shareholder. GK is a cofounder of Samsara Osasuna Therapeutics, everImmune and Therafast Bio. The other authors declare no conflicts of interest.The authors declare that they have no known competing financial interests or personal relationships that could have appeared to influence the work reported in this paper.

## Appendix Supplementary materials

Image, application 1

Image, application 1
