# Supplementary figures and images for: Improved Swiss-rolling method for histological analyses of colon tissue
Source: MethodsX. 2022 Feb 9;9:101630. doi: 10.1016/j.mex.2022.101630 (PMC8861817; doi:10.1016/j.mex.2022.101630)

1. Collect and flush

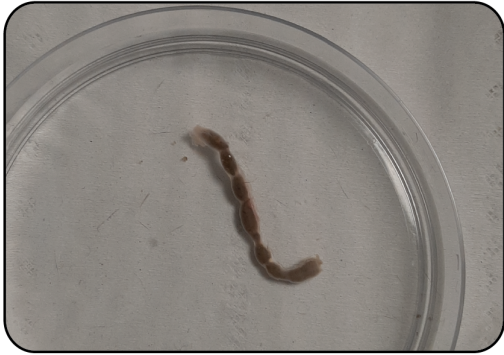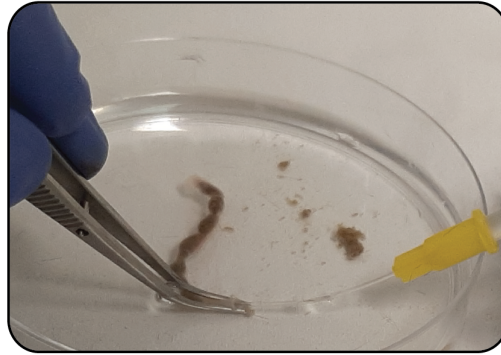

2. Open longitudinally

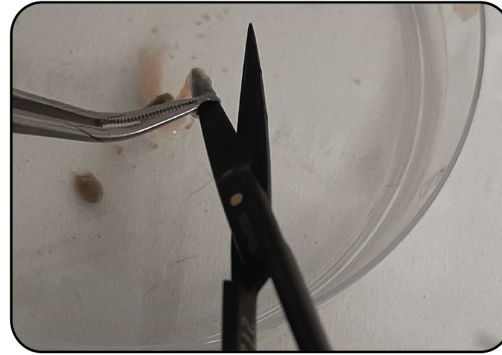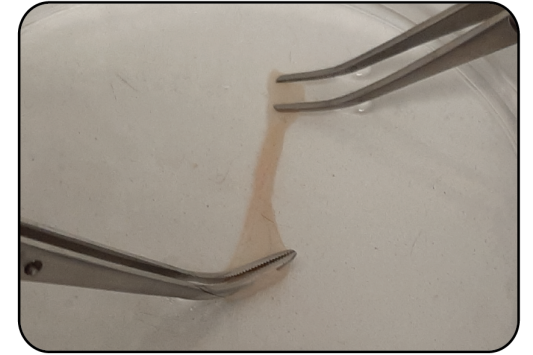

3. Roll

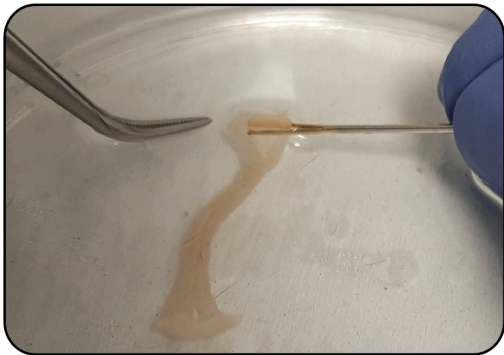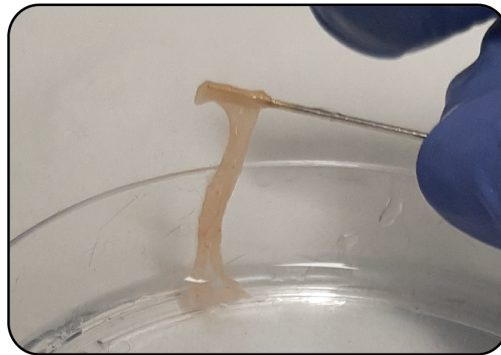

4. Fix with minuten pin

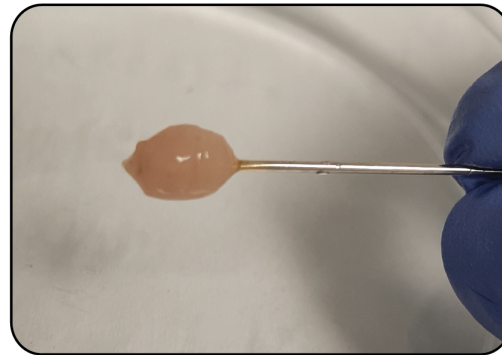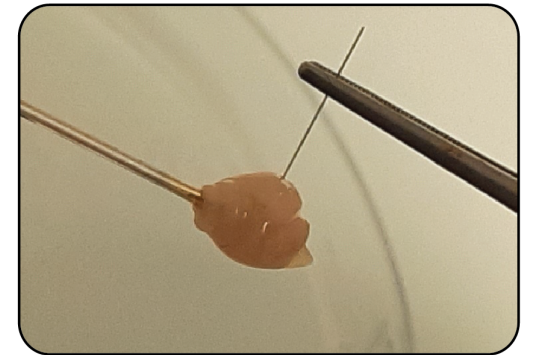

Supplement: Supplementary file 1 [file mmc1.pdf]

**A**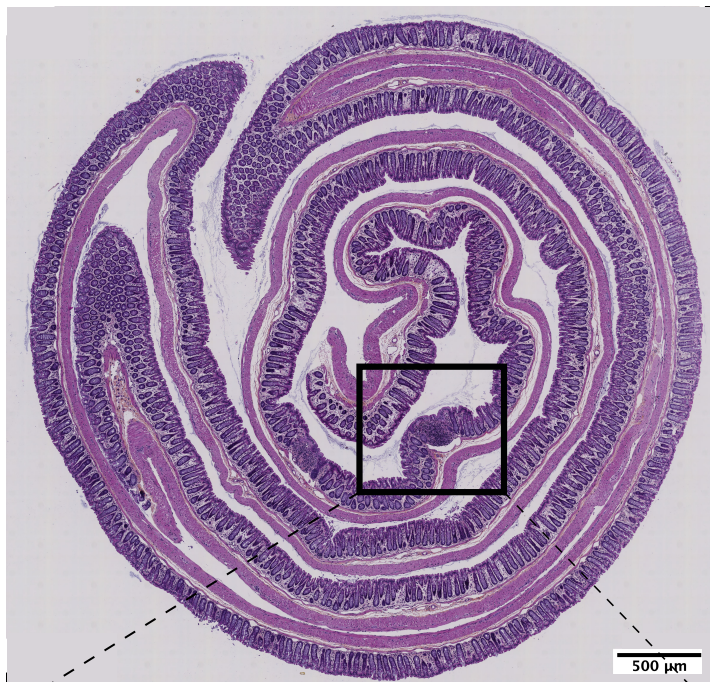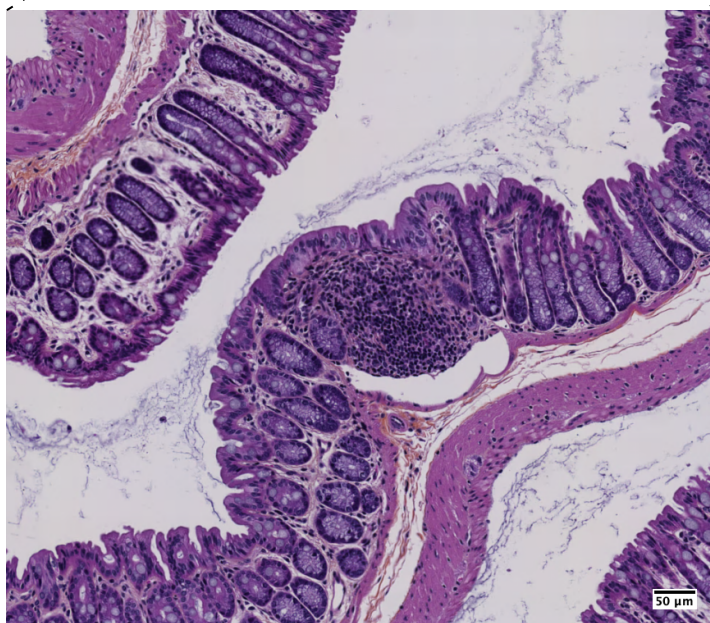**B**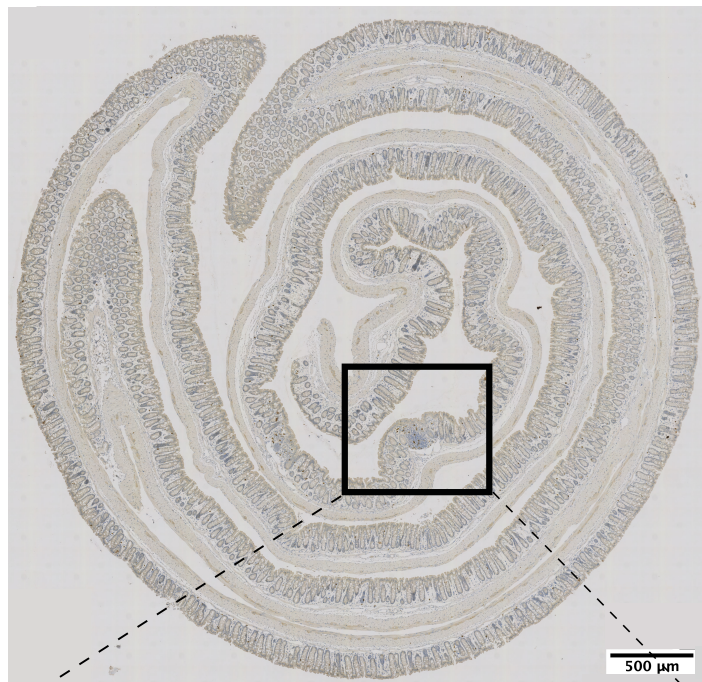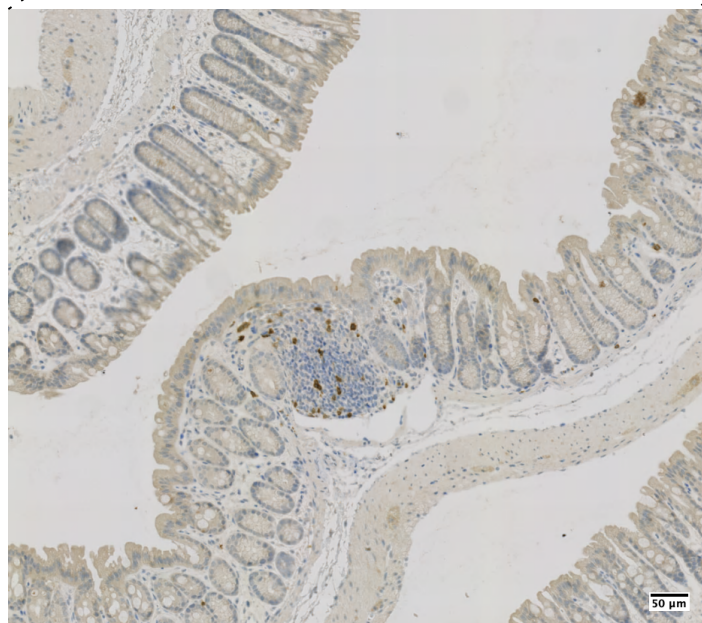

Supplement: Supplementary file 2 [file mmc2.pdf]
